# Unveiling the orchestration: mycobacterial small RNAs as key mediators in host-pathogen interactions

**DOI:** 10.3389/fmicb.2024.1399280

**Published:** 2024-06-05

**Authors:** Rajni Garg, Ishali Manhas, Diksha Chaturvedi

**Affiliations:** ^1^Department of Human Genetics and Molecular Medicine, Amity School of Health Sciences, Amity University, Mohali, Punjab, India; ^2^Department of Biotechnology, Amity School of Biological Sciences, Amity University, Mohali, Punjab, India; ^3^Department of Biotechnology and Medical Engineering, National Institute of Technology, Rourkela, Odisha, India

**Keywords:** tuberculosis, *Mycobacterium tuberculosis*, small RNA, cis-encoded, trans-encoded, therapeutics

## Abstract

Small RNA (sRNA) molecules, a class of non-coding RNAs, have emerged as pivotal players in the regulation of gene expression and cellular processes. *Mycobacterium tuberculosis* and other pathogenic mycobacteria produce diverse small RNA species that modulate bacterial physiology and pathogenesis. Recent advances in RNA sequencing have enabled identification of novel small RNAs and characterization of their regulatory functions. This review discusses the multifaceted roles of bacterial small RNAs, covering their biogenesis, classification, and functional diversity. Small RNAs (sRNAs) play pivotal roles in orchestrating diverse cellular processes, ranging from gene silencing to epigenetic modifications, across a broad spectrum of organisms. While traditionally associated with eukaryotic systems, recent research has unveiled their presence and significance within bacterial domains as well. Unlike their eukaryotic counterparts, which primarily function within the context of RNA interference (RNAi) pathways, bacterial sRNAs predominantly act through base-pairing interactions with target mRNAs, leading to post-transcriptional regulation. This fundamental distinction underscores the necessity of elucidating the unique roles and regulatory mechanisms of bacterial sRNAs in bacterial adaptation and survival. By doing these myriad functions, they regulate bacterial growth, metabolism, virulence, and drug resistance. In *Mycobacterium tuberculosis*, apart from having various roles in the bacillus itself, small RNA molecules have emerged as key regulators of gene expression and mediators of host-pathogen interactions. Understanding sRNA regulatory networks in mycobacteria can drive our understanding of significant role they play in regulating virulence and adaptation to the host environment. Detailed functional characterization of Mtb sRNAs at the host-pathogen interface is required to fully elucidate the complex sRNA-mediated gene regulatory networks deployed by Mtb, to manipulate the host. A deeper understanding of this aspect could pave the development of novel diagnostic and therapeutic strategies for tuberculosis.

## Introduction

Of all the infectious diseases known to mankind, tuberculosis is perhaps the most perplexing one. The discovery of the causative bacterium, *Mycobacterium tuberculosis* (Mtb) as its pathogenic agent by Sir Robert Koch in 1882, was heralded as a breakthrough in medicine. Nearly a century and half later, this infection continues to present a global challenge to healthcare (Comas et al., 2013). Moreover, evolution of this bacterium and excessive use of antibiotics has led to the emergence of Multi Drug Resistant (MDR) and Extensively Drug Resistant (XDR) strains. According to the WHO, TB caused an estimated 1.30 million deaths globally in 2022 (Global Tuberculosis Report, 2023). A lot of research has been done to understand the pathogenesis of Mtb and the mechanisms by which it can evade host defense. The survival and proliferation of Mtb in human macrophages is possible by a complex interplay of virulence factors. The expression of these virulence factors is regulated at transcriptional, post-transcriptional and translational stage. Amongst these, post-transcriptional regulation of gene expression by small RNAs has gained a lot of interest in the past two decades (Haning et al., 2014). Small RNAs (sRNAs) are tiny, versatile, non-coding RNAs which are 50–250 nucleotides long. They are involved in gene regulation by means of RNA interference and modification. sRNAs play a pivotal role in pathogenesis of several bacteria like *E. coli*, *B. subtilis*, and *Salmonella* (Waters and Storz, 2009). sRNAs were first identified in Mtb by northern blotting analysis in 2009 (Arnvig and Young, 2009). Early *in-vitro* research revealed that the expression of sRNAs like B55, F6 and ASpks is seen at low pH and high free fatty acid environment (Lovewell et al., 2016). These conditions mimic the activated macrophage environment and hence, it was postulated that sRNAs might help Mtb to survive the hostile macrophage environment (Vandal et al., 2009). Using high throughput sequencing techniques, many more sRNAs were identified (Arnvig et al., 2011; Ignatov et al., 2012; Pelly et al., 2012). Of late, sRNA Mcr11 has been shown to play a key role in the growth and central metabolism in Mtb (Girardin and McDonough, 2020). In this review, we have summarized the mechanisms of sRNA mediated regulation in Mtb and how they influence mycobacterial pathogenesis. We have also explored new avenues of sRNA secretion into the host that can pave way for novel theranostic approaches for TB that work at host-pathogen interface.

## Bacterial small RNAs

Small regulatory RNAs (sRNAs) are considered to be major gene regulators in bacterial metabolism and survival (Schwenk and Arnvig, 2018). For regulation of gene expression, the majority of sRNAs base-pair with target mRNAs and modulate translation efficiency and mRNA stability (Waters and Storz, 2009; Storz et al., 2011). These sRNAs can be cis-acting if they are present at the same genetic locus or trans-acting if they are present on separate genetic locus. Trans-acting sRNAs are characterized by their involvement in base-pairing interactions with a limited number of RNA molecules and a small subset of protein-binding RNAs like the highly conserved *E. coli* 6S RNAs and CsrB, both of which modify protein activity, by binding to specific proteins (Schwenk and Arnvig, 2018).

## Cis-encoded sRNAs

**Figure 1:**
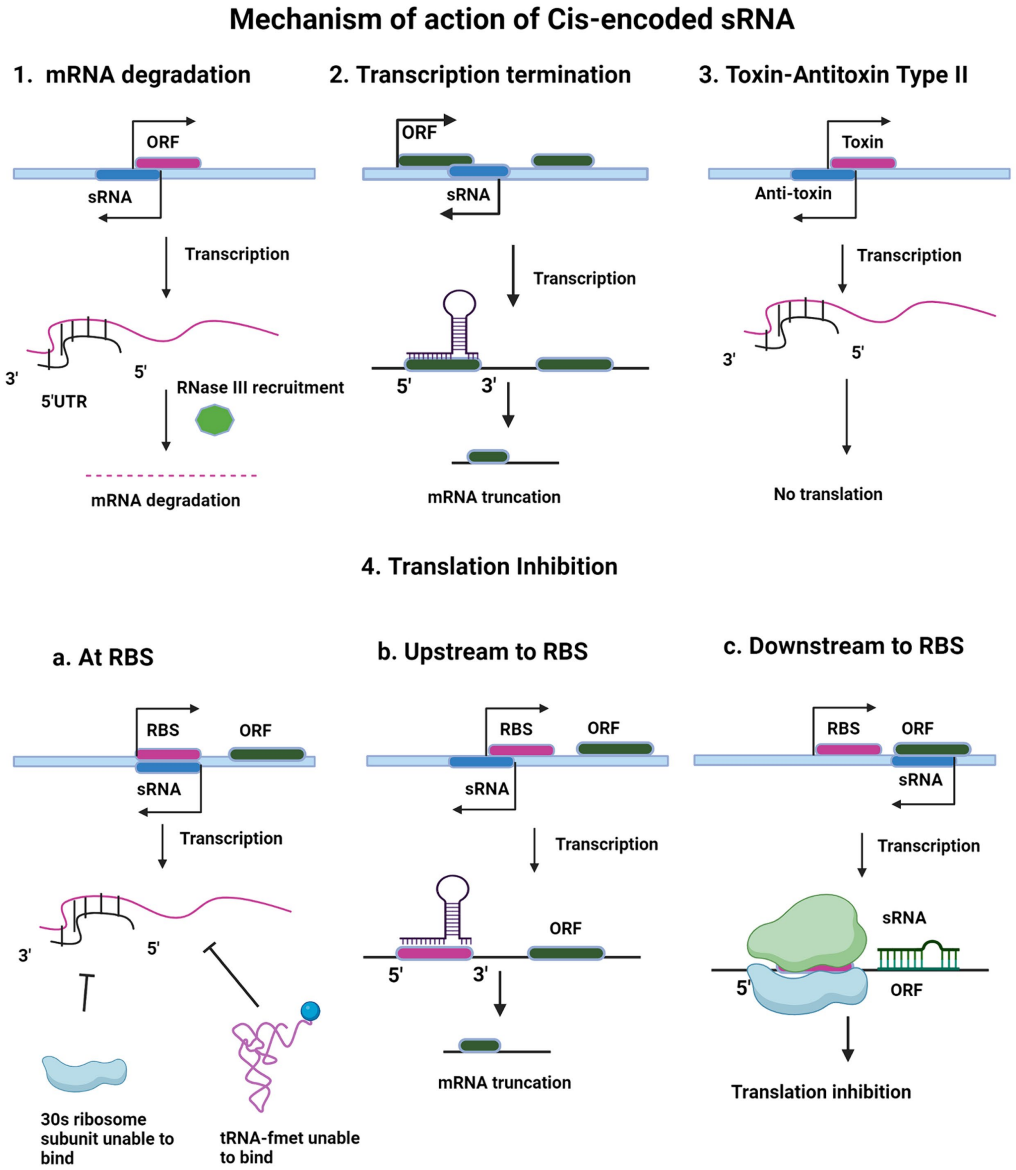
Regulatory mechanism of cis-encoded small RNA. The figure shows different mechanisms of action of cis-encoded sRNA like mRNA degradation (1), transcription termination (2), toxin-antitoxin Type II (3) and translation inhibition (4). In all the mechanisms, sRNA is depicted in blue color. Target ORF is depicted in pink color (1, 2, and 3). Upon intramolecular base pairing interaction of sRNA with its target, the different downstream pathways are shown in the schematic. After active transcription, base pairing occurs between the small RNA and complementary sequence in the 5′ untranslated region and initial segment of the ORF of the emergent mRNA. This RNA–RNA hybridization causes mRNA degradation (1) or premature termination of transcription (2). In toxin-antitoxin type II system (3), after transcription, the antitoxin transcript binds and pairs with the toxin transcript, inhibiting translation of the toxin gene transcript. In translation inhibition mechanism (4), cis-encoded sRNA blocks 30S ribosomal subunit and f-met tRNA binding. RBS sequence (purple) on target messenger RNA, obscuring 30S ribosomal subunit recognition, preventing translation initiation (4a). Open reading frame (ORF) is green. sRNA binds upstream of RBS on target mRNA, leading to truncated transcript (4b). Cis-encoded sRNA blocking of translation elongation is shown in 4c.

Cis-encoded sRNAs are small regulatory RNAs that are encoded on the opposite strand of their target mRNA and therefore, they are also called antisense sRNAs (or asRNAs). These asRNAs have the ability of extensive base-pairing in long regions with their target mRNA, often 75 nucleotides or more (Wagner et al., 2002; Brantl, 2007). Generally, cis-encoded sRNAs are expressed from plasmids, phages and transposons, but chromosomally encoded cis-encoded RNAs have also been discovered (Waters and Storz, 2009). These cis-encoded sRNAs maintain the copy number of the bacteriophages, plasmids, and other mobile genetic elements by preventing the synthesis of replication primer plasmid ColE1, RNA I and translation of transposons, as in the case of Tn10 pOUT RNA (Waters and Storz, 2009). Because of their perfect complementarity with their target mRNAs, true asRNAs, facilitate the recruitment of RNase III for degradation of target mRNA (Schwenk and Arnvig, 2018). Cis-encoded sRNAs are found to function as antitoxins in Type II systems where they code for proteases, which degrade toxin proteins, or they bind to toxin, not allowing it to act on target as in case of Type III A TA systems (Waters and Storz, 2009). Figure 1 illustrates the mechanism of action of cis-encoded sRNAs.

## Trans-encoded sRNAs

Trans-encoded sRNAs are diffusible molecules of around 100 bp and they function in trans by binding to their target mRNA located at a distinct place away from their origin. Thus, the formation of such RNA duplexes is mediated by short and imperfect RNA interactions, usually 6–8 bp short ‘seed sequence’, which is extendable. Those sRNA sequence regions, which are involved in base-pairing and targeting multiple mRNAs, tend to be highly conserved than others, like GcvB sRNAs, of *Salmonella* and *E. coli*; these sRNAs have a conserved region which is G/U rich and which specifically binds to C/A rich region in its target mRNAs (Sharma et al., 2007). Due to this limited complementarity, these trans-encoded sRNAs can target multiple mRNAs (Gottesman and Storz, 2011). These are the widespread type of bacterial sRNAs and are found to be highly expressed in stresses like nutrient deficit, oxidative stress, etc. These sRNAs majorly show a regulatory role in the translation and stability of its target and have found analogous functional characteristics similar to eukaryotic microRNAs (Gottesman, 2005; Aiba, 2007).

In contrast to cis-encoded sRNAs, the absence of perfect complementarity in trans-encoded sRNAs necessitates their dependency on RNA chaperone, Hfq. Hfq facilitates chances of productive interaction between trans encoded RNA with their targets (Brantl, 2007; Waters and Storz, 2009; Vogel and Luisi, 2011). Gram-negative bacteria exclusively require Hfq which enhances base-pairing by binding to both trans-encoded sRNAs and target mRNA (Brennan and Link, 2007). Apart from assisting in function, this RNA binding protein is also involved in providing stability to sRNAs, in mRNA splicing and in decay too (Schwenk and Arnvig, 2018). Hfq requiring bacterial pathogens are found to show reduced virulence on its gene deletion (Chao and Vogel, 2010). These sRNAs are also found to have their internal terminator, uridine-rich end, a necessary element for forming the complex with Hfq (Morita et al., 2005; Ishikawa et al., 2012) There is a varying dependency of many species on Hfq. Bacterial species such as *Salmonella typhimurium* and *E. coli* have sRNAs that are highly dependent on this RNA chaperone, whereas, till now in gram-positive bacteria like *Mycobacterium tuberculosis* Hfq or its homologs have not been identified yet (Jousselin et al., 2009; Oliva et al., 2015), that is why the exact mechanism of how mycobacterial sRNAs interact with their target mRNA is still subject of investigation. Similarly, while *Helicobacter pylori* is a gram-negative bacterium, the identification of Hfq or its homologs in this species remains uncertain. Understanding the intricacies of sRNA-mediated regulation in these organisms necessitates exploring alternative RNA chaperones or distinct regulatory mechanisms that may govern sRNA function in the absence of Hfq. Although, Cold Shock protein A (CspA), another RNA chaperone has its homolog present in Mtb (Arnvig et al., 2011; Caballero et al., 2018), and acts by denaturing the secondary structure of RNA (Jiang et al., 1997).

**Figure 2:**
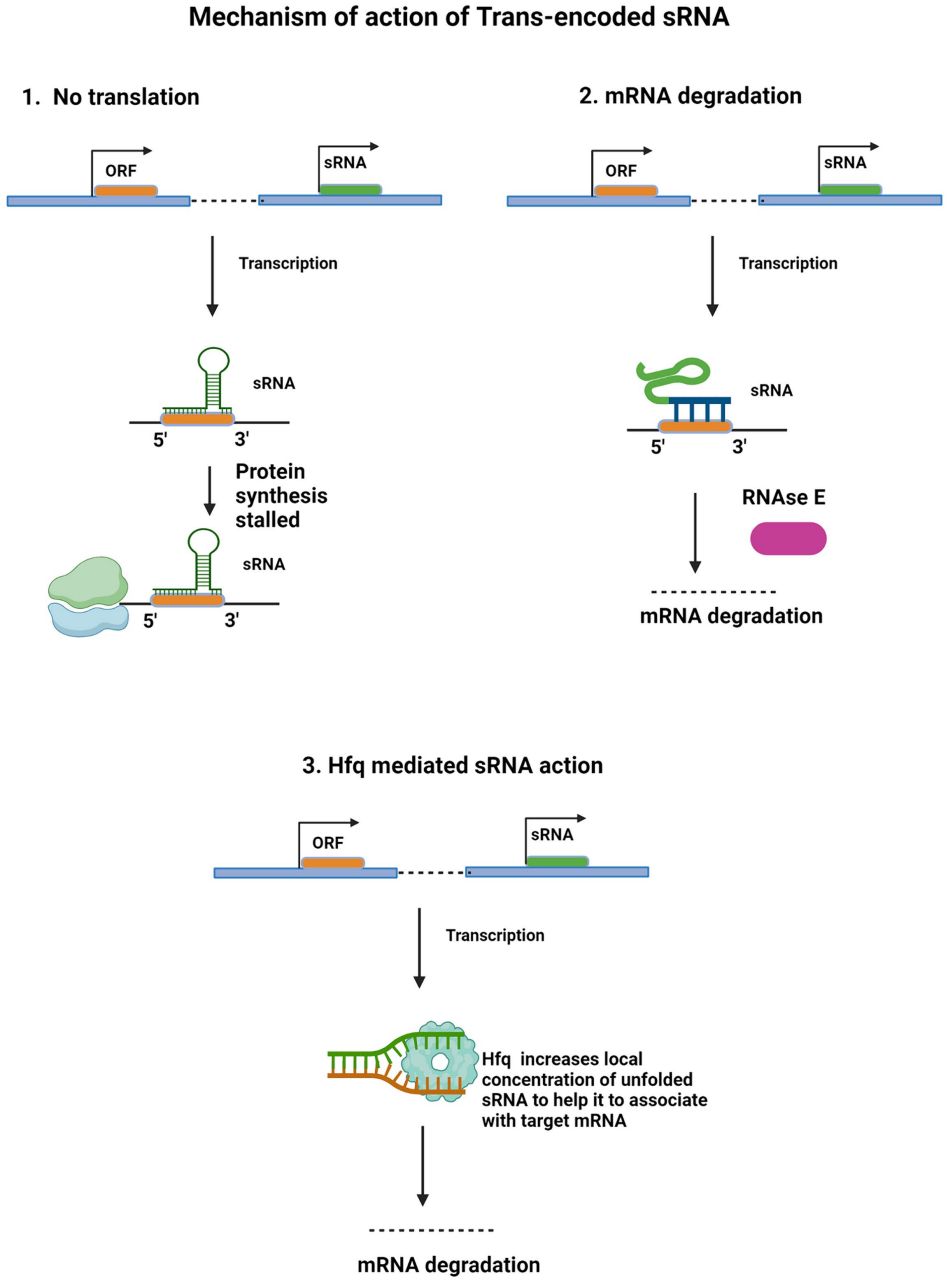
Regulatory mechanism of Trans-encoded sRNA. Trans-encoded sRNA (green) binds to complementary sequence in target mRNA open reading frame (ORF). The RNA duplex interacts with the 30S ribosomal subunit bound to the same mRNA molecule, obstructing translational elongation (1). After independent transcription, partial base pairing occurs between the sRNA and complementary mRNA sequence. This RNA duplex interacts with and recruits RNase E (purple), an endonuclease which cleaves target mRNA, leading to degradation (2). ORF of target mRNA (orange) and regulatory small RNA (green) are independently transcribed. Hfq protein (light blue) binds to and increases sRNA local concentration and availability to pair with target mRNA. By facilitating this base-pairing interaction, Hfq enables sRNA recruitment for mRNA degradation (3).

Structural aspects of base-pairing of trans-encoded sRNAs with their target mRNAs have been studied extensively in *Staphylococcus aureus*, where sRNAs SprD, RNAIII, and RsaE found their target mRNAs by utilizing C-rich regions present in the accessible site of the loop (Geissmann et al., 2009; Bohn et al., 2010; Chabelskaya et al., 2010). Some other sRNAs in *S. aureus* and other bacteria also utilize their C-rich region for recognizing their target mRNAs (Storz et al., 2011). Figure 2 illustrates various mechanisms deployed by trans-encoded sRNAs.

## Mechanism of action of sRNA

### sRNA base-pairing

sRNAs have some specific characteristics which are responsible for their diverse roles (Updegrove et al., 2015). sRNAs genes present within intergenic regions are mostly equipped with Rho-independent terminator containing a stem-region, followed by a poly-U stretch (Chen et al., 2002). This terminator is also found to be resistant to ribonuclease degradation (Ishikawa et al., 2012) and contains an Hfq-binding region (Updegrove et al., 2015).

Studies on cis-encoded sRNAs suggested that base-pairing requires various elements and interactions on several levels (Wagner et al., 2002; Brantl, 2007). The extensive complementary base-pairing between cis-encoded sRNAs and their target mRNAs does not occur immediately across the complementary length but starts with rapid and high-affinity base-pairing with the stem sequence located on the target itself. This initial interaction is referred to as “kissing” interaction (Storz et al., 2011). Following this, base-pairing may extend leading to RNA secondary structure rearrangements (Storz et al., 2011). A new study of the ibsC-SibC mRNA-sRNA duplex in *E. coli* shows the need for various structural elements and interactions at multiple steps (Han et al., 2010).

### sRNA-mediated reduced mRNA stability

Reduction in mRNA stability is also one of the outcomes of sRNA mediated regulation. These sRNA-mRNA duplex formed after base-pairing between cis-or trans-encoded sRNAs and their target mRNAs cause these mRNA to get degraded (Gottesman and Storz, 2011). Trans-encoded sRNA-mRNA duplex is mostly degraded by the endoribonucleases such as RNase E or RNase III (Afonyushkin et al., 2005; Pfeiffer et al., 2009). Mtb has a homolog of both RNase E (present in *E. coli*) and RNase J (present in *B. subtilis*) (Even et al., 2005; Valerio et al., 2011; Durand et al., 2015). These two RNases are more specific for 5′ phosphorylated RNA, and there is a possibility that these RNases have the same function in Mtb as in *E. coli* and *B. subtilis, respectively*, (Mackie, 1998; Koslover et al., 2008; Li de la Sierra-Gallay et al., 2008; Mathy et al., 2010; Valerio et al., 2011). RNase E processes RNAs and causes their degradation (Lu et al., 2014). This endoribonuclease forms a complex called RNA degradosome to degrade mRNAs, in which its C-terminal region is bound with enzymes- polynucleotide phosphorylase (PNPase), enolase, and RNA helicase B (Morita et al., 2005; Carpousis, 2007). Through its C-terminal scaffold region, RNase E is also found forming ribonucleoprotein complexes with Hfq/sRNAs to recruit sRNAs at the target (Lu et al., 2014). Another endoribonuclease, RNase III is involved in regulatory actions of both cis-encoded and trans-encoded sRNAs (Brantl, 2007; Andrade et al., 2012), but the mechanism is still unknown (Lu et al., 2014).

The mechanism of trans-encoded sRNAs causing a reduction in target mRNA stability also involves several exoribonucleases, for instance, PNPase, a 3′-5′ exoribonuclease found to degrade single-stranded RNA and bind to Hfq (Mohanty et al., 2004; Morita et al., 2005). Apart from being involved in RNA degradosome mediated RNA decay, the PNPase also affects the stability of sRNAs such as MicA and RybB without requiring degradosome complex (Andrade and Arraiano, 2008) and is also responsible for turn-over of trans-encoded sRNA, but the mechanism behind it is still unclear (De Lay and Gottesman, 2011; Lu et al., 2014).

### Inhibition of transcription and translation

Many sRNAs act by base-pairing in 5’ UTR region, either at the ribosome binding site (RBS), where they prevent association of the 30S ribosome and fMet-tRNA (Holmqvist, 2012) or at a distant site upstream to RBS and halt translation, by making that site unavailable for ribosome binding (RBS) (Gottesman and Storz, 2011). This sRNA binding region can be at a distance of five codons from the open reading frame as in the case of *Salmonella* RybB sRNA binding to its target ompN mRNA (Bouvier et al., 2008) to 50 or more nucleotides upstream from the RBS (Sharma et al., 2007) where, they may adopt different mechanisms to block ribosome binding (Gottesman and Storz, 2011). In addition to this, RybB sRNA binds to mRNA coding sequence that prevents translation, but the first five codons of this coding sequence, also known as “five codon window”, are required for efficient inhibition of translation.

In some cases, base-pairing at the downstream-regions of the ribosome binding site also occurs in which ribosome binding does not halt (Pfeiffer et al., 2009). This is exemplified by *Salmonella* MicC base-pairing with ompD mRNA at codons 23–26, without RBS (Pfeiffer et al., 2009). MicC sRNA promotes the RNase E activity for the ompD mRNA to get degraded by utilizing endo nucleolytic degradation of mRNA (Pfeiffer et al., 2009).

sRNAs can also cause termination of transcription of distal genes in an operon by recruiting the Rho-transcription factor, if it is bound at 5′ end, possibly by acting co-translationally to inhibit further translation, as found in ChiX sRNA of *Salmonella*, repressing the distal gene in chip cistron (Bossi et al., 2012).

### sRNAs as an activator of transcription and translation

Small RNAs, especially, trans-encoded sRNAs can also function as mRNA translation activators, by preventing the development of inhibitory secondary structure, as its binding to the 5’ UTR region of the target mRNA can stimulate conformational changes which could lead to the availability of RBS to the ribosome and therefore, activate translation (Morfeldt et al., 1995; Majdalani et al., 2005; Prévost et al., 2007; De Lay et al., 2013). This is well-exemplified by the action of the three sRNAs – DsrA, RprA, and ArcZ. Binding of any of these on the inhibiting structure of rpoS mRNA of *E. coli*, would unmask the initiation codon and Shine-Dalgarno sequence from the inhibitory structure of this mRNA, therefore, activate translation (Battesti et al., 2011).

Transcription activation has also been found in RydC sRNA which sequesters site for RNaseE action on cfa1 mRNA of *Salmonella* (De Lay et al., 2013). Other than this, sRNAs have been seen as an activator, because they block the Rho-factor binding site on rpoS mRNA and inhibit its Rho-dependent termination (De Lay et al., 2013). There is also a possibility of this anti-termination by sRNAs binding present in long 5′ leader sequences (Sedlyarova et al., 2016).

### Hfq-mediated sRNA action

The RNA chaperone, Hfq, is a part of the conserved and ubiquitous RNA-binding Sm/Lsm (like-Sm) protein family, which has a role in mRNA degradation and splicing (Brennan and Link, 2007; De Lay et al., 2013). These are known to bind RNA with their ring or doughnut-like multimeric complex, wherein the bacterial Hfq protein is found as homomeric hexamer only (Gottesman and Storz, 2011; De Lay et al., 2013). Hfq performs remodeling of RNA structures without ATP hydrolysis. It facilitates the structural changes in non-coding RNA (Woodson et al., 2018).

Mechanism of interaction of Hfq with RNAs is still not fully clear, but analyses of the crystal structure of the Hfq-RNA complex found role of its structural surface in binding RNA molecules (De Lay et al., 2013). In *S. aureus*, the crystal of Hfq bound with the uridine-rich region of the RNA showed wounding of RNA on the Hfq proximal face in the central hollow region of its ring structure (Schumacher et al., 2002). Whereas in *E. coli*, Hfq crystal with the RNA bound on its Adenine-rich region revealed the distal face opposite to the proximal face (Link et al., 2009).

These trans-encoded sRNAs have a slight secondary intramolecular RNA binding at their 3’-UTR followed by the poly (U) region, which promotes Rho-independent termination of transcription. The initial contact of sRNAs can be at the proximal face itself (Storz et al., 2011; De Lay et al., 2013). There is also an Hfq binding domain adjacent to the poly (U) region (Storz et al., 2011). Other than this, some binding sites for Hfq are also present internally in sRNAs such as DsrA (Brescia et al., 2003), RyhB (Geissmann and Touati, 2004), RybB (Balbontín et al., 2010), and OxyS (Zhang et al., 2002). This internal site of binding for Hfq is further revealed in a study on SgrS (Ishikawa et al., 2012) which requires a U-rich region internally, adjacent to which, is a stem region for efficient Hfq binding (Ishikawa et al., 2012).

The distal face of Hfq has more-affinity for those mRNAs which characteristically have a binding region on ARN motif where, R and N refer to a purine and any base, respectively, as found in DsrA sRNA targeting rpoS mRNA (Soper and Woodson, 2008; Link et al., 2009). Studies found two Hfq binding sites on the rpoS mRNA leader, one in the proximity to the DsrA sRNA binding region and the other in upstream regions (Soper and Woodson, 2008), where the upstream regions having high-affinity for Hfq, enhance base pairing of this sRNA-mRNA duplex but proposed a hypothesis, that the second binding step following the initial base-pairing (De Lay et al., 2013).

Another site on Hfq termed lateral face and also the rim of the hexameric Hfq ring, showed the possibility of having a necessary role in stepwise combining with sRNAs which facilitate binding of mRNA and Hfq dissociation (Sauer et al., 2012). The current hypothesis for revealing the exact mechanism is that Hfq increases local RNA concentration, due to which Hfq needs to specifically target their sRNAs and mRNAs (De Lay et al., 2013). Further, model is proposed that as Hfq may enhance base pairing by performing remodeling of RNA structures as stated earlier (De Lay et al., 2013). The base-pairing begins initially between two hairpin loops or between a loop and a single-strand, which is followed by the base-pairing extension. Hfq facilitates base-pairing with both RNAs forming a ternary complex, which aids in rapid helix –nucleation (Woodson et al., 2018).

## Mycobacterial sRNAs and mechanism behind their regulatory roles

The success of Mtb as a pathogen depends on its capability to adapt to the hostile environment of the macrophage showcasing stresses such as hypoxia, hydrolytic enzymes, antimicrobials, nitric oxide, nutrient limitation, oxidative stress, iron restriction, low pH, membrane stress and reactive oxygen species (Rohde et al., 2007). Within 20 min of phagosytosis by macrophage, Mtb shows differential expression of around 100 genes, which later increase to few hundreds (Rohde et al., 2007). Among Mtb sRNAs, MTS2823, ncRv12659, MrsI, DrrS, and Mcr7 are the most characterized ones (Arnvig et al., 2011; Houghton et al., 2013; Solans et al., 2014; Moores et al., 2017). The various stress conditions encountered by Mtb stimulates production of these sRNAs. Mtb sRNAs are mostly rich in GC content, making them structured to a highly appreciably structured (Schwenk and Arnvig, 2018). These RNAs do not possess usual intrinsic terminator, but possess another type of terminator, referred to as I-shaped terminators within them, which are devoid of uridine-rich 3′-end, unlike intrinsic terminators (Mitra et al., 2009). *In vitro* transcription and RNA seq studies showed insufficiency of I-shaped terminator, to cause transcription termination (Arnvig et al., 2011; Czyz et al., 2014). Because of which generation of 3′ termini occurs by processing in many Mtb sRNAs, unlike many sRNAs, that necessitates a poly-U tail for termination and dependency on Hfq RNA chaperone (Otaka et al., 2011). Table 1 summarizes regulatory roles of various sRNAs in the bacterium and the host.

**Table 1:** List of different mycobacterial small RNAs and their role in Mtb and the host.

S. no.	sRNA	Structure	Role in Mtb	Role in host
1.	MTS1338	108 nt long and a stable secondary structure (Bychenko et al.). Has stem-loop structure	Promotes the expression of operons that cause growth defect in Mtb	Helps Mtb to survive inside the host and enter a dormant state. Induced in presence of NO derivatives, high IFN-γ levels, and low pH
2.	DrrS (DosR regulated small RNA)	Has 5′ stem-loop structure Processed from Drrs+(300 nt) to a stable DrrS (106 nt) (Morgan et al., 1985)	Regulates Rv1734 mRNA stability and gene regulation in response to to NO stress, hypoxia, and stationary phase (Park et al., 2003; Holmqvist and Wagner, 2017).	Accumulates in high levels during chronic infection in mice (Arnvig and Young, 2012). Pathogenicity of Mtb.
3.	MrsI (ncRv11846)	100 nt highly structured sRNA (Shell et al., 2015).	Downregulates the expression of non-essential iron-containing proteins to conserve iron for essential bacterial functions. Downregulates bfrA and fprA (Jacques et al., 2006; Oglesby-Sherrouse and Murphy, 2013).	Induced in the host under iron limiting conditions, oxidative and membrane stress (Richter and Backofen, 2012).
4.	F6	5′ end of the F6 sRNA is highly conserved.	Regulates response to various stresses such as nutrient starvation, cold shock and oxygen depletion hrcA gene (Dar et al., 2016; Holmqvist and Wagner, 2017).	Upregulates genes related to lipid metabolism Acetyl-CoA transferase and Acyl-CoA hydrogenase.
5.	Mcr7	350–400 nt Has extensive folding and a 33-nucleotide long unstructured or free loop (DiChiara et al., 2010).	Targets 18 mRNA molecules including tatC and Rv2053c genes (Romilly et al., 2012; Solans et al., 2014).	By inhibiting the translation of tatC, it impacts the secretion of proteins involved in host-pathogen interactions, virulence, and immune evasion (Solans et al., 2014).
6.	AspkS	75 nt long, during oxidative stress an extended 200 nt AspKs transcript is induced (Arnvig and Young, 2012)	Downregulates pks7, pks8, pks12 and pks15	Found in TB patients’ sera (Fu et al., 2018).
7.	Asdes	Extensive secondary structure with multiple hairpins and internal bulges (Schulze et al., 2016)	Downregulates DesA1	Found in TB patients’ sera (Fu et al., 2018).
9.	As1726	Highly structured sRNA (27 nt)	Regulates tryptophanyl-tRNA synthetase trpS (Arnvig et al., 2011)	Found in TB patients’ sera (Fu et al., 2018).
10.	As1890	Highly structured sRNA (36 nt)	Downregulates Rv1890	Found in TB patients’ sera (Fu et al., 2018).
11.	MTS2823 (ncRv13661)	1st transcript: 300 nt located between genes Rv3661 and Rv3662c. 2nd transcript: ~250 nt appears during the stationary phase (Arnvig et al., 2011).	Overexpression leads to down-regulation of growth related genes. Targets mRNA Rv0115 (hddA), in GDP-L-fucose salvage pathway (Haning et al., 2014).	Found in infected lungs of mice, indicating its potential role in pathogenicity (Haning et al., 2014).
12.	MTS2048 (ncRv12659)	3′ region undergoes processing.	Differential expression of more than 50 genes (Houghton et al., 2013).	5′ region of ncRv12659 present during infection with Mtb (Houghton et al., 2013). Potential biomarker for persisters (Betts et al., 2002; Voskuil et al., 2004; Rustad et al., 2008).

Thus, the multifaceted roles of mycobacterial sRNAs in regulating gene expression underscore their significance in Mtb pathogenesis. Through their diverse mechanisms of action, including transcriptional regulation and mRNA degradation, these sRNAs contribute to the adaptation of Mtb to host stress conditions. Further research into the specific functions of characterized sRNAs, such as DrrS, Mcr7, and MTS2823, promises to unveil novel insights into Mtb-host interactions and potentially inform the development of targeted therapeutic strategies.

## Regulatory roles of sRNA in Mtb

### DrrS (DosR regulated small RNA)

DrrS (DosR regulated small RNA) was first identified in Mtb by RNA-seq (Lamichhane et al., 2013). DrrS is a trans-encoded sRNA, which is generated from precursor transcript, DrrS+ by rapid processing of the 3’end. This mature transcript is 108 nt in length and is highly structured, but its 3′ domain is less-structured which indicates its dependency on Rho-factor (Morgan et al., 1985). A high G:C ratio, a feature of Rho-binding site, appears in a small region of 26 nt only, downstream to 297 nt. Here, the longer transcript does not possess any intrinsic terminator in the initial 350 nucleotides. Collectively, the absence of intrinsic terminator structure, longer transcript DrrS+, and several 3′ termini presents within DrrS^+^ suggests increasing possibility of Rho-dependent transcription termination of DrrS (Moores et al., 2017). Mycobacterial sRNA must go through 3′ processing (Otaka et al., 2011). This processing within DrrS+ is found to be done through the action of mostly both RNase E and RNase J, as Hfq homolog is absent in Mtb. There is a perfect duplex between DrrS^+^ 3′ domain and Rv1734 mRNA, and it may be targeted by RNase III (Moores et al., 2017). It is found that the 5′ stem-loop of DrrS plays a significant role in stabilizing the DrrS, which is reflected by its long half-life. Also, from studies, it is strongly indicated that the 5′ phosphorylation state of recombinant variants of DrrS is found to be modified by RNase phosphohydrolase (RppH) homolog in *M. smegmatis*. Accumulation of DrrS sRNA is dependent on response regulator DosR. Since the sigma factor is expressed on DosR induction, this indicates that DrrS core promoter gets activated by the DosR by an unknown mechanism without requiring the DosR binding site (DBS). Research indicates that dosR gene activates the DrrS core promoter to a notable degree, but in certain tested conditions, both known DBS and the suspected DBS on the upstream region were found to reduce its expression (Moores et al., 2017). DosR is specifically induced during NO stress, hypoxic conditions and to a lesser extent in stationary phase and upregulates around 48 genes which help Mtb to adapt in the host (Park et al., 2003; Holmqvist and Wagner, 2017).

### Mcr7

Mcr7 was found to be a greatly structured sRNA with extensive folding and a 33-nucleotide long unstructured or free loop, as predicted by RNAfold server. Mcr7 is a 350–400 nt, well-studied sRNA and is conserved in *M. tuberculosis* complex (DiChiara et al., 2010). High levels of Mcr7 are found in *M. tuberculosis* H37Rv through RNA-seq studies (Arnvig et al., 2011). It has been studied that Mcr7 targets 18 mRNA where 5′-end region shows complementarity. In some cases, Mcr7 interacts with its 33-nt loop (Romilly et al., 2012). Mcr7 basepairs with its unstructured part with its targets: tatC and Rv2053c genes (Solans et al., 2014). Mcr7 has complementary regions at its 5′-end for the tatC mRNA. These regions include the putativeribosome-binding site (RBS) and initial 6 codons of tatC mRNA. Binding of Mcr7 results in masking of RBS and halting of tatC mRNA translation. In Mtb, the tatC gene encodes a protein that is a transmembrane component of the TatABC secretory apparatus and is needed for translocation of secreted proteins from the cytoplasm to the extracellular environment with a twin-arginine or Arg-Arg (RR) motif present in their signal peptide, which are recognized by the TatC, before export through the TatA channel (Solans et al., 2014).

### MTS2823 (ncRv13661)

MTS2823 is a highly abundant sRNA, around 300-nucleotide long transcript, expressed in exponential and stationary phase cultures (Haning et al., 2014). The Northern blot analysis showed one another form of MTS2823 transcript, around 250-nucleotides long during stationary phase (Haning et al., 2014). It is flanked by genes Rv3661 and Rv3662c. One of its target mRNA is Rv0115 (hddA), coding for D-alpha-D-heptose-7-phosphate kinase which has a role in the GDP-L-fucose salvage pathway. MTS2838 has also been found in infected lungs of mice, which indicates its role in pathogenicity (Haning et al., 2014).

The highest expression of MTS2823 sRNA takes place in stationary phase (approximately 10 fold more than in exponential phase) and adversely affects growth rate of Mtb (Arnvig et al., 2011; Ami et al., 2020). The genes associated with growth in exponential phases are down-regulated upon overexpression of MTS2823 (Arnvig et al., 2011). In Mtb during the exponential phase, 17% of the total non-rRNA are encoded from intergenic regions and referred to as sRNAs, whereas this amount increases to about 60% during the stationary phase, which is comparable to rRNAs, owing to the accumulation of MTS2823, a highly abundant sRNA. Higher than this amount, found in mice, having a chronic infection (Arnvig et al., 2011). Overexpression of its target mRNA Rv0115 causes downregulation of several genes by ≥2.5 fold and some of these targets are Rv3828c, hemD, mpt83, Rv0875, Rv3839, and ribH (Arnvig et al., 2011). It also decreases expression of methyl citrate synthase by around 15 fold (Haning et al., 2014). Overexpression of MTS2823 also leads to upregulation of two genes, Rv2035, encoding HspG activator (upto 3.2-fold) and Rv3229c, a fatty acyl desaturase DesA3 (up to 3.1-fold) (Arnvig et al., 2011; Haning et al., 2014). MTS2823 is also a functional homolog of 6S RNA, although its mechanism is still not clear (Arnvig et al., 2011; Haning et al., 2014). Downregulation of bacterial replication genes is mediated by transcriptional interference by 6S RNA expression, where RNA polymerase associated with principal sigma factor performed the transcription (Trotochaud and Wassarman, 2005). Genes downregulated because of overexpression of MTS2823 are prpC, ppdK, glcB, prpD, lrpG, icl, Rv0465c, Rv1128c, and Rv1126c, where prpC and prpD are methyl citrate genes and lrpG is their regulator, and others are their neighboring genes. Those which are downregulated by 2–2.5-fold are Rv1132, Rv1627c, polA, Rv3075c, acn, pckA, gka2, Rv0843, citA, acs and ltp3. The downregulation of methyl citrate gene prpC is because of the downregulation of several sigma factors, caused by overexpression of MTS2823 (Manganelli et al., 2001; Sun et al., 2004; Arnvig et al., 2011). The overexpression of the MTS2823 downregulated VapC toxin, whereas antitoxin partners did not seem to be affected.

Through STRING database analysis, an extended network of methyl citrate genes was seen getting down-regulated. The downregulation of specific genes such as prpC in particular and to an extent prpD indicates preferential targeting by MTS2823 to decrease either the utilization of propionyl-CoA and/or oxaloacetate or accumulation of toxic intermediates like methyl citrate (Arnvig et al., 2011). On the contrary to Hfq-dependent sRNAs, which degrades along with their target, MTS2823 appears to accumulate in stationary phase, and also during chronic tuberculosis infection in lungs of mice (Arnvig et al., 2011).

### MrsI (ncRv11846)

Mycobacterial regulatory sRNA in iron (MrsI) or ncRv11846 is a trans-encoded, 100 nt long highly structured sRNA. Its transcription start site (TSS) is around 100 nt upstream of an unannotated gene, Rv1847 (Shell et al., 2015). It also has a predicted rho-independent terminator at its 3′ end (Czyz et al., 2014). The mycobacterial iron-dependent transcription factor, IdeR has a binding site near the TSS of MrsI in Mtb and *M. smegmatis* (Prakash et al., 2005) which could be the reason for the increased amount of this sRNA in iron-limiting conditions, and probably its involvement in iron-limiting response of bacteria (Gerrick et al., 2018). MrsI has bacterioferritin (bfrA), as its target mRNA, which is regulated by direct binding interaction. MrsI has a short seed sequence of 6–8 nt which interact with the target mRNA and has 7-nt apical single-stranded loop. This was tested by transcriptional profiling in *M. smegmatis* during iron-limitation conditions, which identifies 20 MrsI regulated genes found in higher levels in *mrsI* deletion strain on comparing with wild-type ones. These 20 genes were assembled in 12 transcripts, and out of which, 8 were found to encode for nonessential proteins which play a role in iron metabolism which includes proteins as the bacterioferritin BfrA, the [NiFe] hydrogenase maturation factor HypF and ferredoxin reductase FprA (Gerrick et al., 2018).

Studies conducted on *M. smegmatis*, by fusing luciferase reporter gene to the MrsI promoter, found high levels induction in iron deprivation and not in other conditions such as oxidative stress and membrane stress in Mtb. bfrA and fprA are found to be regulated in Mtb as well. In Mtb, transcriptional profiling studies, combined with the CRISPR mediated knockdown of the sRNA, resulted in an abundance of 118 genes, and out of these, 106 were associated with iron deprivation, 12 with membrane stress and rest 5 were associated with oxidative stress (Gerrick et al., 2018).

### MTS2048 (ncRv12659)

MTS2048 (ncRv12659) overlaps with the open reading frame of Rv2660c. It is the most up-regulated RNA in Mtb during starvation and is present at an adjacent site to the PhiRv2 prophage. The presence of only one Transcription start site (TSS) and no apparent terminators, led to the assumption that ncRv12659 is processed from the longer transcript (Houghton et al., 2013). Since TSS of MTS2048 is present within the PhiRv2 prophage, it may be concluded that only strains containing PhiRv2 exhibit sRNA expression (Houghton et al., 2013). High levels of MTS2048 cause growth defect in Mtb H37Rv. During starvation, an integrase promoter Rv2659c activates the sRNA promoter which indicates a repressive impact that triggers the phage lytic cycle (Houghton et al., 2013).

### F6

5’end of sRNA F6 is highly conserved within *Mycobacterium* species (Arnvig and Young, 2012; Dar et al., 2016). It is highly induced during nutrient starvation, cold shock, oxygen depletion and repressed during heat shock conditions (Dar et al., 2016; Holmqvist and Wagner, 2017). Overexpression of F6 extremely slows down Mtb growth rate (Arnvig and Young, 2009) of Mtb. Comparative phenotypic and expression analysis between F6 WT, deletion and complemented Mtb strains revealed a relation between nutrient starvation and F6 expression (Houghton et al., 2013). During acid stress, the expression of F6 is induced by 2 fold and when Mtb is nutrient starved by static incubation for 96 h, F6 expression increases by >75 fold (Arnvig and Young, 2009). It specifically regulates expression of HrcA heat shock repressor in host. HrcA regulates expression of heat shock inducible chaperonins like Rv0440, Rv3418c and conserved hypothetical protein coding genes Rv0991c, Rv0990c (Stewart et al., 2003). Sigma factors regulate many genes involved in stress adaptations in Mtb during persistence state (Hu et al., 2000). F6 promoter contains a SigF promoter motif, which when overexpressed effects the growth rate of cells (Houghton et al., 2013). Anaerobiosis also induced expression of SigF followed by induced expression of sRNA F6 (Hu et al., 2000). When bacteria are given heat shock, SigF is downregulated.

In conclusion, exploring regulatory roles of small RNAs (sRNAs) in orchestrating gene expression dynamics contributes to our understanding of bacterial adaptation within the host environment. Through their diverse mechanisms of action, sRNAs such as DrrS, Mcr7, and MTS2823 play pivotal roles in fine-tuning the response of Mtb to various stress conditions encountered during infection. The identification of target mRNAs and the elucidation of sRNA-mediated regulatory networks provide valuable insights into the molecular mechanisms underlying Mtb pathogenesis. Continued research into the functional characterization of Mtb sRNAs promises to uncover novel regulatory pathways and potential therapeutic targets for combating tuberculosis. The next section discusses regulatory role of mycobacterial sRNAs in the host to facilitate the same.

## Regulatory roles of mycobacterial sRNAs in the host

**Figure 3:**
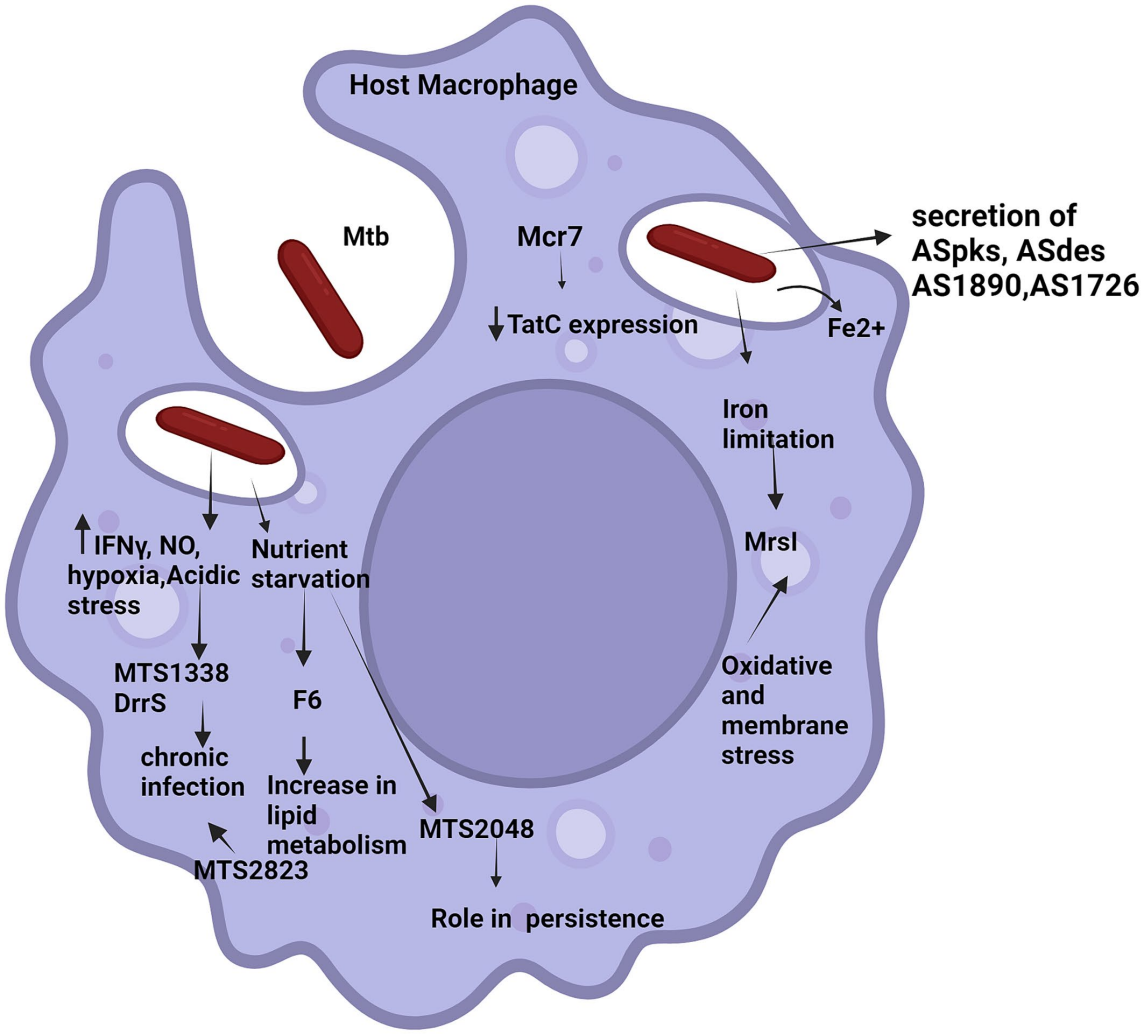
Potential roles of regulatory mycobacterial small RNAs in the host. The figure illustrates various external signals triggering small RNAs in host (various small RNAs have been represented by names). Solid arrows up/down indicate increase/decrease in the expression level of the molecules, respectively. Tapered arrows indicate downstream steps of different pathways.

Many pathogenic bacteria use sRNA molecules to regulate cell cycle events, modulate their response in stressed conditions and thus virulence (Arnvig and Young, 2009, 2012; Haning et al., 2014). Apart from the role of sRNA in Mtb in the previous section, we will also describe role of some identified cis-encoded sRNA transcripts are ASpks, desA1, pks12, AS1726, and AS1890c in the host in this section (Betts et al., 2002; Arnvig et al., 2011). sRNA molecules regulate the expression of target mRNA. Research is being done to experimentally validate the associated roles of sRNAs in enhancing or repressing translation of mRNA targets (Betts et al., 2002). The following section will elaborate role of sRNAs identified in Mtb in host pathogenesis. Figure 3 shows diagrammatic representation of potential roles of regulatory mycobacterial small RNAs in the host.

### DrrS

The presence of a stem loop structure at 5′ end makes DrrS a very stable sRNA, as this does not allow mycobacterial RppH homolog to bind and protects the 5′ end from RNAse activity. The levels of DrrS+ is highest during Mtb early stationary phase and DrrS108 accumulates during the late stationary phase at least up to 3 weeks (Moores et al., 2017) marking longevity in infection. The role of DrrS is dependent upon its processing. Both DrrS+ and DrrS108 have different regulons and hence different functions (Eruslanov et al., 2004). This understanding of stabilized regulation of mRNA can be applied to manipulate expression levels of different proteins in biological systems and to optimize immune system for vaccine development (Rohde et al., 2007). DrrS accumulates in high levels in mice during chronic infection, indicating its importance in achieving dormancy in the host (Arnvig and Young, 2012).

### MTS1338

Overexpression of trans-encoded MTS1338 helps Mtb to survive inside the host and attain dormancy which is followed by reactivation of the pathogen when the host immune system is down (Stewart et al., 2003). It was reported that mimicking host conditions like using nitric oxide derivatives, high IFN-γ levels and low pH induce expression of MTS1338 in mycobacterial cultures (Holmqvist and Wagner, 2017; Hör et al., 2018). Mycobacterial sRNA, regulatory proteins, and their cognate target proteins in Mtb work together to subvert the host immune system (Ignatov et al., 2015; Dutta and Srivastava, 2018). Lowering the pH enhanced the growth of the overexpression strain suggesting that overexpression of MTS1338 makes cells more resistant to acidic pH (Hu et al., 2000; Betts et al., 2002). *In vivo* studies with low doses of wild type Mtb caused relatively faster infection spread in I/St mice, which produce less IFN-γ than in B6 mice. It was found that 10 weeks post infection, MTS1338 expression in I/St mice was higher than in B6 mice (Ignatov et al., 2015; Dutta and Srivastava, 2018). Increased MTS1338 synthesis causes the metabolism to gradually shut down, which is one of strategies of Mtb for assisting its survival inside the host. Additionally, MTS1338 was expressed ten times more in IFN-γ activated macrophages than in control cells (Majorov et al., 2003). MTS1338 expression is largely triggered by NO, even in the absence of IFN-γ activation. This was discovered when MTS1338 expression could not be increased by IFN-γ stimulated macrophages, treated with L-NIL (NO synthase inhibitor) (Stewart et al., 2003). MTS1338-induced genes contributed to Mtb survival in oxygen- and energy-deficient environments. Down-regulated genes were found to be participating in translation, cell replication and biogenesis (Lovewell et al., 2016). These changes in transcriptional landscape induced by MTS1338 help the pathogen to reduce its metabolic activities and enter a dormant state.

### MrsI (ncRv11846)

As discussed in the previous section, MrsI is expressed in *in vitro* cultures under iron-limiting conditions (Gerrick et al., 2018). It is a small independent transcript which downregulates its target regulon (bfrA/fprA) by directly binding to its 5’ UTR region of mRNA and eventually degrading them. bfrA and fprA code for iron-containing proteins. When MrsI represses its regulons, it represses the expression of non-essential iron-containing proteins hence retaining iron for essential Mycobacterium functions. Rapid repression of bfrA is observed when Mtb is pre-exposed to oxidative and membrane stress before iron-deprivation. This may be an anticipatory response of Mtb to prepare itself for impending iron-deficiency. Mtb may use oxidative stress and membrane stress as the signals of warning that the host macrophage will soon get deprived of iron, and thus it should start saving iron for its essential protein functions and enter an iron-sparing state. Thus, MrsI helps Mtb to anticipate iron deprived conditions, adapt, and survive in host macrophage.

### MTS2048 (ncRv12659)

MTS2048 shows high levels of induction occurs during infection with PhiRv2 positive strain of Mtb, and this acts as a potential biomarker for identifying cells undergoing both starvation and hypoxia which later might turn into persisters (Betts et al., 2002; Voskuil et al., 2004; Rustad et al., 2008). It has also been observed that only a 5′ region of MTS2048 is present during infection with Mtb, which suggests transcription termination or 3′ end processing (Houghton et al., 2013). Differential expression of more than 50 genes was observed after overexpression of this sRNA, where PhiRv2 was found to be on top of the list (Houghton et al., 2013).

### F6

F6 helps Mtb in intracellular survival within the host by upregulating genes coding for enzymes Acetyl-CoA transferase and Acyl-CoA hydrogenase which play an important role in lipid metabolism (Lovewell et al., 2016). Anaerobiosis conditions show induced F6 expression and Mtb transitions to persistent phase. The measured respiration rates show that ΔF6 strain is impaired while complement strain displays intermediate phenotype. This shows that F6 might have some role in reviving non replicating Mtb cultures hence intensifying host’s later stages of infection (Lamichhane et al., 2013; Ignatov et al., 2015). As mice do not form granulomas, they do not generate a strong phenotype change. Thus, for this C3HeB/FeJ strain of mouse can be used as they have the ability to form lung granulomas (Houghton et al., 2013).

In summary, mycobacterial sRNAs play pivotal roles in modulating host immune responses and influencing the outcome of Mtb infection. By fine-tuning the expression of virulence factors and immune evasion strategies, these regulatory molecules shape the host-pathogen interaction landscape. The detection of mycobacterial sRNAs in TB patients underscores their clinical relevance and potential as diagnostic markers. Moving forward, elucidating the intricate interplay between sRNAs and host immunity holds promise for the development of innovative therapeutic interventions targeting Mtb infections.

## Mycobacterial sRNAs detected in TB patients

### ASpks

ASpks an antisense transcript that aligns with the mRNA, is a 75 nucleotide long transcript (Arnvig and Young, 2012), encoding duplicate identical ketosynthase domains within the pks12 gene in *M. tuberculosis*. The pks12 gene is involved in the synthesis of mannosyl-b-1-phosphomycoketide molecules, which are recognized as antigens by CD1-restricted T cells. ASpks has the potential to act as both a cis-encoded and trans-encoded sRNA, depending on the boundaries of the sRNA. ASpks shows significant complementarity to regions within pks7, pks8, and pks15, suggesting a potential regulatory role in the expression of these genes involved in polyketide synthesis (Arnvig and Young, 2009). ASpks was detected in the serum samples of active tuberculosis patients (Fu et al., 2018).

### ASdes

ASdes can act as both a cis-encoded and trans-encoded sRNA, regulating the expression of desA1 and desA2 (Arnvig and Young, 2009). It shows significant complementarity to desA1 (Rv0824c), a fatty acid desaturase essential for the growth of Mtb and is upregulated during infection. ASdes also aligns with desA2 (Rv1094), another desaturase gene, indicating a potential regulatory role in lipid metabolism. It was detected in the serum of tuberculosis patients (Fu et al., 2018).

### AS1890 and AS1726

AS1890 is a small RNA identified in *M. tuberculosis* with a size of 36 nucleotides (Arnvig and Young, 2009). AS1726 is another small RNA identified in *M. tuberculosis* with a size of 27 nucleotides (Arnvig and Young, 2009). These small RNAs (AS1726 and AS1890) were also found in the serum of patients, suggesting their presence and possible involvement in *Mycobacterium tuberculosis* infection (Fu et al., 2018).

To summarize, the identification of mycobacterial sRNAs in TB patients highlights their potential as diagnostic biomarkers for disease detection and monitoring. These sRNAs exhibit differential expression patterns in response to Mtb infection, suggesting their involvement in host-pathogen interactions. Leveraging these sRNAs as diagnostic tools may aid in early detection and treatment of TB, ultimately contributing to improved patient outcomes and disease management.

## Mycobacterial sRNAs role in host immune response and therapeutic targeting

Mycobacterial small RNAs (sRNAs) play a multifaceted role in shaping the host immune response during infection. These small regulatory molecules play a crucial role in modulating the production of virulence factors that are critical for mycobacterial pathogenicity and survival in the host environment. Targeting particular genes and immune response-related pathways, sRNAs can precisely regulate immune signaling cascades, affecting the host’s capacity to identify and eliminate the invasive pathogen. Furthermore, it has been shown that mycobacterial sRNAs modify the gene expression associated with immune evasion tactics, allowing the pathogen to evade host immune surveillance and establish persistent infections. Through their regulatory functions, sRNAs can orchestrate a delicate balance between pro-inflammatory and anti-inflammatory responses, shaping the overall immune landscape during mycobacterial infection. Insights gained from studying the role of sRNAs in host immune responses not only deepen our understanding of mycobacterial infections but also hold potential for the development of novel therapeutic interventions (Haning et al., 2014). Some mycobacterial sRNAs may target host immune cell functions, such as macrophage activation or cytokine production, to subvert the host immune response and promote bacterial persistence (Coskun et al., 2021). The potential therapeutic targeting of mycobacterial small RNAs (sRNAs) presents a promising avenue for future medical interventions. One approach involves the development of antibiotics that specifically target sRNA-enabled virulence mechanisms, offering a novel way to disarm pathogens and overcome microbial resistance to traditional antimicrobials. Understanding the functions of small RNAs in modulating gene expression in mycobacteria opens up possibilities for RNA-based therapeutics that can disrupt pathogenic processes and enhance treatment outcomes. By harnessing the regulatory power of sRNAs, researchers seek to advance precision medicine approaches that target the molecular mechanisms driving mycobacterial infections, paving the way for more effective and tailored treatment options in the fight against these challenging diseases (Haning et al., 2014). Advancements in technologies such as CRISPR interference-based assays and locked nucleic acid (LNA) power inhibitors provide tools for studying and manipulating sRNAs, paving the way for the development of novel therapeutic strategies targeting mycobacterial sRNAs (Gerrick et al., 2018).

## Conclusion

In conclusion, it can be said that small RNAs in Mtb form an intricate network of regulatory molecules that play a crucial role in bacterial adaptation, virulence, and pathogenesis. The ability of Mtb to survive and thrive within the hostile environment of the macrophage is facilitated by the differential expression of numerous small RNAs in response to various stress conditions such as hypoxia, nutrient limitation, oxidative stress, and increase in IFNγ. These small RNAs, including MTS2823, MTS2048, MrsI, DrrS, and Mcr7, have been identified as key players in the regulatory machinery of Mtb, responding to cues of the host and modulating gene expression for adaptation in the host. Small RNAs like MTS1338, MTS2823 and DrrS have role in chronic infection, while MTS2048 has a role in persistence (Betts et al., 2002; Voskuil et al., 2004; Rustad et al., 2008). The structural characteristics of Mtb small RNAs, such as their high GC content and unique terminator sequences, highlight their specialized mechanisms of action and processing. For instance, the DosR-regulated small RNA DrrS undergoes rapid processing at its 3′ end to generate a mature, highly structured transcript that is dependent on factors like Rho for termination. The intricate interplay between small RNAs, transcriptional regulators, and mRNA targets underscores the sophisticated regulatory landscape of Mtb. By targeting specific small RNAs involved in virulence or antibiotic resistance, researchers may uncover new avenues for combating drug-resistant strains of Mtb and improving treatment outcomes for tuberculosis patients. Moreover, the insights gained from studying small RNA networks in Mtb could have broader implications for unraveling similar regulatory mechanisms in other bacterial pathogens, paving the way for innovative approaches to combat infectious diseases.

## Future directions

The study of small RNAs in Mtb holds significant promise for future implications in the field of tuberculosis research, diagnostics, and therapeutics. By delving deeper into the regulatory roles of small RNAs in Mtb and their interactions with host cells, researchers can uncover novel avenues. Small RNAs in Mtb that are crucial for virulence and pathogenesis could serve as potential targets for novel therapeutic interventions. By developing small molecule inhibitors or antisense oligonucleotides that specifically target these regulatory molecules, researchers may be able to disrupt essential pathways in Mtb that are necessary for survival in the host. By targeting key components of the small RNA machinery, researchers may discover innovative approaches to combat drug-resistant strains of Mtb and enhance the efficacy of existing antibiotics. Few sRNA (described in the previous section) have been detected in patients’ sera. By identifying unique small RNA signatures associated with Mtb infection, researchers could develop sensitive and specific diagnostic tests for early detection of the disease, leading to improved patient outcomes and better disease management. The underexplored area is regulation of host genes by these secreted sRNA. By elucidating how small RNAs manipulate host gene expression, researchers may uncover new targets for host-directed therapies that enhance the immune response against Mtb infection. By profiling small RNA expression patterns in individual patients, clinicians may be able to tailor treatment regimens to target specific vulnerabilities in the bacterial population, leading to more effective and personalized therapies.

Emerging technologies in RNA sequencing, such as single-cell RNA sequencing (scRNA-seq) and long-read sequencing platforms, offer unprecedented opportunities to dissect the complex regulatory networks of small RNAs in Mtb. scRNA-seq can provide insights into the heterogeneity of bacterial populations within host tissues, shedding light on how small RNAs contribute to mycobacterial adaptation and persistence (Han et al., 2015). Long-read sequencing technologies enable the comprehensive characterization of small RNA structures and interactions, enhancing our understanding of sRNA-mediated gene regulation in Mtb (Han et al., 2015). Furthermore, the integration of RNA sequencing technologies into therapeutic development pipelines presents a promising avenue for novel treatment strategies targeting small RNAs in Mtb. By leveraging RNA sequencing data to identify key regulatory sRNAs involved in virulence, antibiotic resistance, and pathogenesis, researchers can design precision therapies that specifically target these molecules. Small molecule inhibitors or antisense oligonucleotides tailored to disrupt essential pathways in Mtb could lead to the development of innovative anti-TB treatments, particularly against drug-resistant strains. Additionally, the application of RNA sequencing in identifying unique small RNA signatures associated with Mtb infection in patients’ sera holds potential for the development of sensitive and specific diagnostic tests for early disease detection, ultimately improving patient outcomes and disease management. By harnessing the capabilities of RNA sequencing technologies in therapeutic research, researchers can unlock new avenues for combating tuberculosis effectively and advancing precision medicine approaches tailored to individual patients. This forward-looking approach not only deepens our understanding of small RNA networks in Mtb but also paves the way for transformative advancements in TB treatment and control.

## Author contributions

RG: Conceptualization, Formal analysis, Supervision, Writing – original draft, Writing – review & editing. IM: Investigation, Writing – original draft. DC: Data curation, Writing – review & editing.
